# The Association of Wagner Classification, Microbial Resistance, Immune Markers, and Glycemic Control With Diabetic Foot Ulcer Severity: A Multi-disciplinary Approach to Predict Severity and Outcome

**DOI:** 10.7759/cureus.90914

**Published:** 2025-08-24

**Authors:** Muhammad Numan Saleem, Awal Mir, Umair Ullah, Muhammad Talha, Haq Nawaz, Abdur Rehman, Roh Ullah

**Affiliations:** 1 Internal Medicine, Hayatabad Medical Complex Peshawar, Peshawar, PAK; 2 Hematology, Khyber Medical University, Peshawar, PAK; 3 Institute of Paramedical Sciences, Khyber Medical University, Peshawar, PAK

**Keywords:** diabetic foot infection, glycemic control, immune markers, microbial sensitivity, wagner classification

## Abstract

Background: Leukocytes play a crucial role in immune function, and inflammatory markers such as neutrophil-to-lymphocyte ratio (NLR), platelet-to-lymphocyte ratio (PLR), and monocyte-to-lymphocyte ratio (MLR) are emerging as important predictors of diabetic foot ulcer (DFU) severity, infection risk, and healing outcomes. These markers may aid in early detection and improved prognosis. This study aims to evaluate the predictive value of inflammatory markers (NLR, PLR, MLR, total leukocyte count (TLC), absolute lymphocyte count (ALC)) in relation to DFU severity, glycemic control, and healing outcomes.

Methodologies: A comparative cross-sectional study was conducted at the endocrinology Ward of Hayatabad Medical Complex (HMC), Peshawar. A total of 102 diabetic foot ulcer patients were included in the study. Patients' consent, demographic data, and clinical data were taken. Blood samples were collected for hematological, biochemical analysis, and wound culture samples for bacterial species identification and drug sensitivity. Data was collected and analyzed through SPSS 22 (IBM Corp., Armonk, NY, USA).

Results: A weak negative correlation was observed between ALC and Wagner grade (ρ = -0.134), which was not statistically significant. TLC also demonstrated a weak negative correlation with Wagner grade (ρ = -0.240), approaching but not reaching statistical significance. A weak positive correlation was found between TLC and hemoglobin A1C (HbA1C) (ρ = 0.199), which was statistically significant. In contrast, ALC showed no significant correlation with either HbA1c (ρ = -0.002) or fasting blood sugar (FBS) (ρ = -0.066). Statistically significant reductions were observed in FBS (p < 0.001), random blood sugar (p < 0.001), and TLC (p < 0.001) following treatment. Microbiological analysis of diabetic foot ulcers revealed a diverse range of bacterial isolates. The most frequently identified organism was *Pseudomonas aeruginosa* (13, 12.7%), followed by *Escherichia coli* (12, 11.8%) and *Proteus* species (11, 10.8%).

Conclusions: No significant association was observed between ALC and Wagner grade in this study. Further studies with larger sample sizes are needed to clarify this relationship. However, a positive correlation between TLC and glycemic control suggests that poor glycemic control can affect the immune system and decrease the healing process, which may result in increased hospital stays for patients or unhealed ulcers leading to amputation.

## Introduction

Diabetic foot is one of the most common and severe complications of diabetes. It is composed of deep tissue lesions, mostly caused by damaged nerves and peripheral vascular disease in the lower extremities [1]. The worldwide prevalence of diabetic foot is estimated to be 6.3% [2]. In Pakistan, the combined prevalence of diabetic foot ulcer (DFU) is 12.16% [3]. About 5% of diabetic patients develop a foot ulcer each year, and around 15% of all diabetes patients face this problem at some point during their illness [4]. DFUs impose a significant clinical and economic burden on healthcare systems worldwide, leading to a marked decrease in the quality of life for affected individuals [5]. The leading cause of hospitalization among diabetes patients is DFU and amputation [6].

People with diabetes are at a higher risk of developing foot infections because of various underlying factors, including impaired immune function, peripheral vascular disease, peripheral neuropathy, trauma, and infection [7]. Due to a lack of feeling, minor foot harm from things like badly fitted shoes or injuries goes undetected. This type of trauma can cause ulceration and subsequent infection if it is repeated or ignored [8]. A range of cells, such as mast cells, neutrophils, lymphocytes, monocytes, macrophages, keratinocytes, fibroblasts, and endothelial cells, are involved in and contribute to the distinct stages of skin wound repair [9]. Lymphocytes are an important part of the adaptive immune system that play a vital role in maintaining normal immune function [10]. The balance between the production and death of these cells is carefully regulated by the body [11]. Lymphocytes are crucial in the healing process because they produce cytokines and growth factors, known as lymphokines. They also significantly influence fibroblast activity and the formation of new tissue during wound healing. The T cell-dependent immune system actively contributes to wound healing, and its depletion can impair this process. In individuals with type 2 diabetes and diabetic foot ulcers, the rate of T cell death (apoptosis) is significantly higher compared to those with type 2 diabetes but no ulcers. Increased oxidative stress in diabetics with chronic, non-healing wounds triggers lymphocyte apoptosis, further complicating the healing process [12].

Diabetic foot ulcer severity can be assessed by the Wagner classification, which is the most commonly used system for assessing diabetic foot ulcers. This classification focuses on ulcer depth and bone involvement in DFU [13]. The Wagner classification, originally described by Meggitt in 1976 and later popularized by Wagner in 1981, categorizes diabetic foot ulcers based on wound depth. The system categorizes ulcers into six levels. Grade 0 means the skin is unbroken. Grade 1 is a shallow ulcer on the surface. Grade 2 is when the ulcer goes deeper, reaching tendons, bones, or joints. Grade 3 involves a severe ulcer with an abscess or infected bone. Grade 4 is when gangrene develops in the forefoot, and grade 5 is when the gangrene spreads to the whole foot [14].

Diabetic foot infections (DFIs) can host a diverse range of pathogens [15]. DFIs may involve either a single microorganism or multiple pathogens, with polymicrobial infections being more frequently observed in chronic cases that have been previously treated with antibiotics [16]. In a 2017 study, Ullah et al. found Escherichia coli (19, 19%), Staphylococcus aureus (nine, 9%), and Pseudomonas (seven, 7%) as the most prevalent bacteria in DFU [17].

Several inflammatory and immune markers, including the neutrophil-to-lymphocyte ratio (NLR), red cell distribution width (RDW), platelet-to-lymphocyte ratio (PLR), and monocyte-to-lymphocyte ratio (MLR), have been identified as useful indicators for predicting the occurrence, prognosis, and outcomes of DFU. Growing evidence indicates that these markers are crucial for the early detection of DFUs, potentially enhancing patient outcomes [18]. These studies have revealed a significant decrease in lymphocyte count following the DFU severity. However, there is a lack of research to explore the direct relationship between absolute lymphocyte count and the severity of DFUs classified by Wagner grade, particularly regarding glycemic control, which could provide more simpler and cheaper way to predict early detection of DFU severity. Absolute lymphocyte count (ALC) could be used as a cheaper prognostic marker. Additionally, while it's well established that glycemic control influences immune function and wound healing in diabetic patients, the possible relationship between blood sugar levels, lymphocyte count, and the severity of diabetic foot ulcers as categorized by the Wagner classification has not been extensively explored [19]. This study had three primary objectives. First, to evaluate whether ALC correlates with DFU severity (Wagner classification). Second, to examine links between immune markers (total leukocyte count (TLC), ALC, platelet count (PLT)) and glycemic control (hemoglobin A1C (HbA1C), fasting blood sugar (FBS)). Third, to identify prevalent bacterial pathogens and their antibiotic resistance patterns in DFUs. We hypothesized that lower ALC and higher TLC would associate with advanced Wagner grades and poor glycemic control, respectively.

## Materials and methods

A comparative cross-sectional study was conducted at the endocrinology ward at Hayatabad Medical Complex (HMC) and Institute of Paramedical Sciences, Khyber Medical University, Peshawar, Pakistan. A convenient sampling technique was used to collect data from all admitted patients in the endocrinology ward at HMC from July 7 to December 1, 2024. Patients aged 18 years or older, diagnosed with DFU, who had culture and sensitivity reports, pre- and post-treatment data, and Wagner classification assessments were included in the study. Patients with incomplete clinical records or missing laboratory data (complete blood count (CBC), random blood sugar (RBS), FBS, HbA1c, wound culture), those who did not provide consent or were unable to participate in both pre- and post-treatment data collection, and pediatric patients. A total of 102 were studied according to online sample size calculator for descriptive studies (OpenEpi; https://www.openepi.com/SampleSize/SSPropor.htm).

Samples were collected for hematological, biochemical analysis, and wound culture to assess bacterial growth and antimicrobial susceptibility. CBC was performed using an automated hematology analyzer (XN-1000; Sysmex, Kobe, Japan), following manufacturer protocols and daily internal quality control procedures. RBS, FBS, and HbA1c levels were analyzed using an automated biochemistry analyzer (Cobas 6000; Roche Diagnostics, Indianapolis, IN, USA), with both internal and external quality controls in place to ensure accuracy and precision. Wound swab samples were collected aseptically and cultured on selective and non-selective media including blood agar, MacConkey agar, and chocolate agar. Plates were incubated aerobically at 37°C for 18-24 hours. Bacterial isolates were identified based on colony morphology, Gram staining, and standard biochemical tests such as catalase, coagulase, oxidase, and API 20E where applicable. Antimicrobial susceptibility testing was performed using the disc diffusion method (Kirby-Bauer) on Mueller-Hinton agar, following Clinical and Laboratory Standards Institute guidelines. Quality control for culture and sensitivity was ensured using reference strains such as Escherichia coli ATCC 25922 and Staphylococcus aureus ATCC 25923.

In addition to these tests, various comorbidities were assessed in diabetic foot ulcer patients, as prolonged high glucose levels can cause damage to multiple organs. The comorbidities assessed included retinopathy, congestive heart disease, and chronic kidney failure. The Wagner classification data were also recorded for each patient to assess the severity of DFU based on the depth of the ulcer, presence of infection, and extent of tissue involvement. This grading system helped categorize the patients’ ulcers from grade 0 to 5 for clinical correlation. Grade 0 indicates no ulceration, although foot deformities or signs of inflammation may be present. Grade 1 refers to an ulcer confined to the superficial layer of the skin without involvement of deeper tissues. Grade 2 describes an ulcer that extends into subcutaneous structures such as tendons, ligaments, or fascia. Grade 3 involves a deep ulcer accompanied by infection, abscess formation, or bone involvement. Grade 4 is characterized by localized gangrene affecting part of the forefoot, while Grade 5 represents extensive gangrene involving most or the entire foot [12].

Test data were collected in two phases: pre-treatment and post-treatment. This was done to observe any improvements after the patients were admitted to the hospital for diabetic control and ulcer wound healing. In hematological tests, CBC was performed to assess various parameters in correlation with the Wagner classification. For example, lymphocyte count was evaluated to different grades of Wagner classification for DFU, and neutrophil count was assessed in connection with the severity of infection.

In biochemical tests, RBS, FBS, and HbA1c levels were measured. RBS is used to check blood glucose levels at any random time during the day. The normal blood glucose level for a healthy person is around 100 mg/dL. On the other hand, FBS measures blood glucose levels after at least eight hours of fasting, with a normal range of 70-100 mg/dL. The HbA1c test checks average glucose levels over the past three months to determine whether glucose levels have been maintained within the normal range. This test can also help identify whether a person is pre-diabetic, diabetic, or non-diabetic. These biochemical tests were conducted to evaluate the severity of DFU based on RBS, FBS, and HbA1c levels.

A wound culture sensitivity test was performed to check for bacterial contamination at the ulcer site. In this test, a wound swab was gently collected from the ulcer area of the diabetic foot and placed in a sterile tube with the lid properly closed. The swab was then sent to the microbiology section of the laboratory for culture to identify microbial growth at the ulcer site. In the culture sensitivity test, the antibiotic sensitivity of these microbes was also assessed. The antibiotics to which the microbes were sensitive were then prescribed to patients to reduce the infection.

## Results

A total of 102 patients (both male and female) were enrolled in the study. Among them, 63 (61.8%) were males and 39 (38.2%) were females, with a mean age of 56.75 ± 10.24 years. The mean duration of diabetes was 15.81 years, and all patients were diagnosed with type 2 diabetes mellitus. Based on severity, 63 (61.8%) patients had severe diabetes, 19 (18.6%) had moderate diabetes, and 20 (19.6%) had mild diabetes. Detailed distribution of diabetes severity, comorbidities, and complications is presented in Table 1.

**Table 1:** Descriptive analysis and frequency distribution of Wagner classification among diabetic foot patients All values are presented as frequency and percentage N (%). Abbreviations: IHD = Ischemic Heart Disease, PVD = Peripheral Vascular Disease, HTN = Hypertension, HCV = Hepatitis C Virus, CVA = Cerebrovascular Accident.

Parameters	Sub Parameters	Frequencies
Diabetes Severity (HbA1c %)	Mild	20 (19.6%)
	moderate	19 (8.6%)
	severe	63 (61.8%)
Gender (Years)	Male	63 (61.8%)
	Female	39 (38.2%)
Diabetes Complications	Retinopathy	57 (55.9%)
	IHD	3 (2.9%)
	PVD	1 (1.00%)
	No complications	22 (21.6%)
	multiple complications	19 (19.9%)
Co-Morbidities	No comorbidities	74 (72.5%)
	Sepsis	1 (1.00%)
	HTN	21 (20.6%)
	HCV	2 (2.00%)
	CVA	3 (2.9%)
	Ascites	1 (1.00%)
Wagner Classification	Grade 1	24 (23.50%)
	Grade 2	8 (7.80%)
	Grade 3	30 (29.40%)
	Grade 4	28 (27.50%)
	Grade 5	12 (11.80%)

Most patients presented with moderate to severe diabetic foot ulcers, with the highest frequencies observed in Wagner Grade were 30 (29.4%) and Grade 4 were 28 (27.5%). Fewer patients were classified under early-stage (Grade 1) or very severe (Grade 5) ulcers. For a comprehensive overview of descriptive statistics, laboratory findings, and related analyses, refer to Table 1 and Table 2.

**Table 2:** Descriptive statistics of age, duration of diabetes, and hematological parameters before and after treatment All values are expressed as range and mean ± standard deviation (SD). Abbreviations: FBS = Fasting Blood Sugar, RBS = Random Blood Sugar, TLC = Total Leukocyte Count, ALC = Absolute Lymphocyte Count, PLT = Platelet Count, HB = Hemoglobin, HbA1c = Glycated Hemoglobin.

Parameter	Pre-Treatment		Post-Treatment		
Name (units)	Range	Mean ± SD	Range	Mean ± SD	
FBS (mg/dL)	Up to 461	251.44 ± 11.02	Up to 495	170.54 ± 79.75	
RBS (mg/dL)	Up to 545	270.67 ± 12.25	Up to 441	214.35 ± 83.55	
TLC (×10³/µL)	Up to 25.08	14.11 ± 5.81	Up to 22.82	12.39 ± 5.18	
ALC (×10³/µL)	Up to 7.03	2.13 ± 1.11	Up to 6.26	2.06 ± 0.97	
PLT (×10³/µL)	Up to 829	372.23 ± 15.72	Up to 750	387.15 ± 14.38	
HB (g/dL)	Up to 11.55	10.20 ± 2.07	Up to 8.4	10.34 ± 1.74	
HbA1c (%)	Up to 10.048	9.29 ± 3.62	Up to 8.6	8.2±2.71	
Diabetes Duration (years)	Up to 34	15.81 ± 7.27	Up to 37	17.23 ± 8.01	
Age (years)	Up to 53	56.75 ± 10.24	Up to 53	56.75 ± 10.24	

Relationship between absolute lymphocyte count and Wagner classification

The Spearman’s rank correlation analysis was conducted to evaluate the relationships between absolute lymphocyte count and Wagner grade, other key clinical variables, including diabetes duration, FBS, TLC, PLT, and HbA1c. Correlation with Wagner grade was also evaluated.

A weak negative correlation was observed between ALC and Wagner grade (ρ = -0.134, p = 0.349), which was not statistically significant. TLC showed a weak negative correlation with Wagner grade (ρ = -0.240, p = 0.090), nearing significance but not reaching the threshold for statistical significance. No significant correlations were found between diabetes duration, pre-FBS, or HbA1c and Wagner grade, with all p-values exceeding 0.05. Table 3 shows the detailed relationship between Wagner, ALC, and other variables.

**Table 3:** The associations between absolute lymphocyte count, Wagner grade, and other key clinical variables. Abbreviations: FBS = Fasting Blood Sugar, TLC = Total Leukocyte Count, ALC = Absolute Lymphocyte Count, PLT = Platelet Count, HbA1c = Glycated Hemoglobin. ρ = Spearman’s correlation coefficient. “p-value” indicates statistical significance level.

Variable	ρ (Correlation with Wagner Grade)	p-value (Sig.)	Interpretation
Diabetes Duration (years)	0.192	0.178	Weak positive
FBS	0.002	0.989	No correlation
TLC	-0.24	0.09	Weak negative
ALC	-0.134	0.349	Weak negative
PLT	-0.251	0.075	Weak negative
HbA1c	-0.021	0.884	No correlation

Evaluation of immune markers and their association with glycemic control

Spearman’s rank correlation analysis was performed to explore the relationship between immune markers (TLC, ALC, PLT) and glycemic control parameters (HbA1c, FBS) in diabetic foot ulcer patients.

A weak positive correlation was observed between TLC and HbA1c (ρ = 0.199, p = 0.048), which was statistically significant. ALC showed no significant correlation with HbA1c (ρ = -0.002, p = 0.985) or FBS (ρ = -0.066, p = 0.509). PLT did not demonstrate any significant correlation with HbA1c (ρ = -0.054, p = 0.597) or FBS (ρ = -0.020, p = 0.840). No significant correlations were found between FBS and any of the immune markers, including TLC (ρ = -0.023, p = 0.818), ALC, or PLT. Table 4 explains this relationship in detail.

**Table 4:** The association between immune markers (TLC, ALC, PLT) and glycemic control parameters (HbA1c, FBS) Abbreviations: TLC = Total Leukocyte Count, ALC = Absolute Lymphocyte Count, PLT = Platelet Count, FBS = Fasting Blood Sugar, HbA1c = Glycated Hemoglobin. ρ = Spearman’s correlation coefficient. “p-value” indicates statistical significance level.

Variable Pair	ρ (Correlation Coefficient)	p-value (Sig.)	Interpretation
TLC and HbA1c	0.199	0.048	Weak positive (Significant)
ALC and HbA1c	-0.002	0.985	No correlation
PLT and HbA1c	-0.054	0.597	No correlation
TLC and FBS	-0.023	0.818	No correlation
ALC and FBS	-0.066	0.509	No correlation
PLT and FBS	-0.02	0.84	No correlation

Changes in immune and inflammatory markers pre-treatment and post-treatment

Paired sample t-tests were conducted to evaluate changes in FBS, RBS, TLC, ALC, hemoglobin (HB), and PLT before and after treatment.

The results showed statistically significant reductions in FBS (p < 0.001), RBS (p < 0.001), and TLC (p < 0.001) following treatment. These results indicate that the treatment was effective in reducing both glycemic markers and the inflammatory marker TLC. However, no significant changes were observed in ALC (p = 0.466), HB (p = 0.434), or PLT (p = 0.175). These markers did not show meaningful differences before and after treatment (Table 5).

**Table 5:** Summary of paired sample t-test results for glycemic and hematological markers before and after treatment. Abbreviations: FBS = Fasting Blood Sugar, RBS = Random Blood Sugar, TLC = Total Leukocyte Count, ALC = Absolute Lymphocyte Count, PLT = Platelet Count, HB = Hemoglobin, Std Dev = Standard Deviation, Std Error = Standard Error, df = degrees of freedom

Pair		Mean Diff	Std Dev	Std Error	t	df	p-value
Pre FBS - Post FBS	80.9		111.56	11.05	7.32	101	0
Pre RBS - Post RBS		56.31	143.86	14.24	3.95	101	0
Pre TLC - Post TLC		1.78	4.86	0.48	3.68	100	0
Pre ALC - Post ALC		0.08	1.17	0.12	0.73	100	0.46
Pre HB - Post HB		-0.15	1.92	0.19	-0.79	100	0.43
Pre PLT - Post PLT		-17.29	127.18	12.66	-1.37	100	0.17

Bacterial isolates in diabetic foot ulcers and their antibiotic sensitivity and resistance profiles

**Figure 1:**
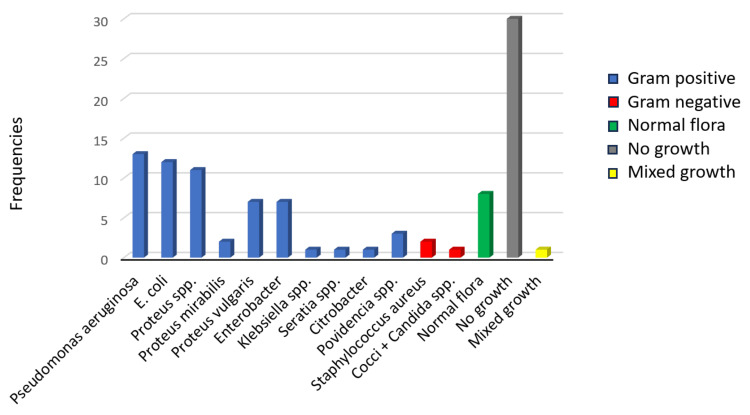
This figure illustrates the frequency of various bacterial isolates identified from diabetic foot ulcer samples

The bacterial isolates from diabetic foot ulcers revealed a diverse range of microorganisms. The most frequently isolated bacterium was Pseudomonas aeruginosa (13, 12.7%), followed by E. coli (12, 11.8%) and Proteus spp. (11, 10.8%). Other notable isolates included Proteus mirabilis (two, 2.0%), Proteus vulgaris (seven, 6.9%), and Enterobacter (seven, 6.9%). Staphylococcus aureus was isolated in two (2.0%) cases, while normal flora was present in eight (7.8%) of the samples. A significant portion of the samples (30, 29.4%) showed no bacterial growth, suggesting either the effectiveness of prior antibiotic treatments or the presence of non-bacterial infections. There was also a single case of mixed bacterial growth (one, 1.0%), indicating polymicrobial infections. Lesser isolates included Citrobacter (one, 1.0%), Seratia spp. (one, 1.0%), and Klebsiella spp. (one, 1.0%). The cumulative percentage showed that the first few isolates, particularly P. aeruginosa and E. coli, accounted for a substantial proportion of the total bacterial growth, reflecting their common association with diabetic foot infections. The results underscore the presence of both gram-negative and gram-positive bacteria, which could impact treatment strategies (Figure 1).

The antibiotic sensitivity and resistance patterns revealed variable efficacy across the tested agents. Meropenem (43, 83.7%), imipenem (50, 74.0%), and piperacillin-tazobactam (54, 72.2%) exhibited the highest sensitivity rates, making them the most effective antibiotics in this study. Clindamycin and vancomycin both demonstrated 100% sensitivity, although they were each tested against only two isolates.

Colistin (43, 67.4%), polymyxin B (47, 68.1%), and gentamicin (50, 64.0%) showed intermediate sensitivity levels, with corresponding resistance rates of 32.6%, 31.9%, and 36.0%, respectively. Tigecycline (49, 63.3%) and amikacin (54, 68.5%) also exhibited moderate effectiveness.

On the other hand, co-amoxiclav showed the highest resistance rate (45, 86.5%), followed by cefotaxime (34, 77.3%) and ciprofloxacin (34, 65.4%). Cefazoline (two, 66.7%) and ceftriaxone (five, 62.5%) also demonstrated limited efficacy. Ceftazidime (46, 56.5%) and cefepime (53, 54.7%) showed moderate sensitivity rates.

See Table 6 for a detailed analysis of the antibiotic sensitivity and resistance percentage to all tested organisms. Among the bacterial isolates, Pseudomonas aeruginosa (13 isolates), Escherichia coli (12 isolates), and Proteus spp. (11 isolates) were the most frequently identified.

**Table 6:** Total percentage sensitivity and resistance of commonly used antibiotics against all tested organisms. Abbreviations: N = Number of isolates tested; Co-Amoxiclav = Amoxicillin + Clavulanic Acid; “–” indicates not applicable or not tested for resistance.

Antibiotic	N	Sensitive (%)	Resistant (%)
Amikacin	54	37 (68.5%)	17 (31.5%)
Ciprofloxacin	52	18 (34.6%)	34 (65.4%)
Colistin	43	29 (67.4%)	14 (32.6%)
Gentamicin	50	32 (64.0%)	18 (36.0%)
Imipenem	50	37 (74.0%)	12 (24.0%)
Meropenem	43	36 (83.7%)	6 (14.0%)
Piperacillin + Tazobactam	54	39 (72.2%)	15 (27.8%)
Polymyxin B	47	32 (68.1%)	15 (31.9%)
Tigecycline	49	31 (63.3%)	18 (36.7%)
Ceftazidime	46	26 (56.5%)	20 (43.5%)
Cefoperazone	40	29 (72.5%)	11 (27.5%)
Sulbactam	11	7 (63.6%)	3 (27.3%)
Co-Amoxiclav	52	7 (13.5%)	45 (86.5%)
Clindamycin	2	2 (100%)	-
Cefepime	53	29 (54.7%)	24 (45.3%)
Cefotaxime	44	10 (22.7%)	34 (77.3%)
Vancomycin	2	2 (100%)	-
Ceftriaxone	8	3 (37.5%)	5 (62.5%)
Peropenem	3	2 (66.7%)	1 (33.3%)
Ampicillin	1	1 (100%)	-
Ceftaroline	3	1 (33.3%)	2 (66.7%)
Erythromycin	2	1 (50.0%)	1 (50.0%)

P. aeruginosa showed resistance to ciprofloxacin in seven (53.8%) isolates and gentamicin in six (46.2%) isolates, while it exhibited complete sensitivity to colistin in 13 (100%) isolates and high sensitivity to polymyxin B in 12 (92.3%) isolates. E. coli demonstrated resistance to ciprofloxacin and imipenem in 10 (83.3%) isolates each, and to meropenem in 11 (91.7%) isolates. However, it was fully sensitive to tigecycline in 12 (100%) isolates and moderately sensitive to polymyxin B in nine (75.0%) isolates. Proteus spp. showed moderate sensitivity to amikacin and gentamicin in seven (63.6%) isolates each, while it exhibited resistance to imipenem in eight (72.7%) isolates and to ceftriaxone in 10 (90.9%) isolates.

## Discussion

The primary aim of this study was to evaluate the relationship between ALC and the severity of diabetic foot ulcers, classified by Wagner grade. In addition, the study sought to explore the association of these immune markers with glycemic control parameters (HbA1c, FBS) and to assess the impact of treatment on these markers. Finally, bacterial isolates in diabetic foot ulcers and their antibiotic resistance profiles were examined to provide insights into effective treatment strategies.

A weak negative correlation was found between ALC and DFU severity classified by Wagner grade, which was not statistically significant (p = 0.349).

This finding suggests that although ALC may play a role in the severity of diabetic foot but the relationship which this study found is very weak and also not significant so we can’t suggest ALC as a reliable marker for Wagner grade to predict DFU severity. In the existing studies, MLR, NLR and PLR have been shown as reliable markers to assess DFU severity [18]. Zhang et al. also found a negative correlation between ALC and Wagner grade, but they suggested PLR as a prognostic marker for DFU severity [20]. Our study suggests that we can’t use ALC alone as a marker for diabetic foot severity classified by Wagner grade. The inflammatory response in diabetic foot ulcers is multifactorial and may not be solely dependent on immune markers like lymphocyte count [21].

There was a significant positive correlation (p = 0.048) between TLC and HbA1c, which suggests that uncontrolled high glucose levels for an extended period may result in immune dysregulation. This may result in unhealed DFU, which is consistent with prior research [22]. However, ALC and platelets showed no significant correlations with HbA1c or FBS, which contradicts some studies that suggest an association between low ALC and poor glycemic control. The lack of significant correlations between ALC, platelets, and glycemia in our study may be due to the relatively small sample size, which can reduce statistical power [23].

There was a significant reduction in FBS, RBS, and TLC following treatment. This indicates that glycemic control and inflammation were effectively managed through treatment. However, no significant changes were detected in ALC, hemoglobin, or platelet count, which suggests that while treatment was effective in reducing glycemic and inflammatory markers, it may not have had a substantial impact on specific immune markers like ALC [24].

The most important finding of this study was identifying the change in the shift of bacterial prevalence. A diverse range of microorganisms was identified in this study from the bacterial isolates of diabetic foot ulcer, with Pseudomonas aeruginosa (13, 12.7%), Escherichia coli (12, 11.8%), and Proteus spp. (11, 10.8%) being the most frequently identified. These findings contrast with a previous study conducted in the same region by Ullah et al. (2017), who found Escherichia coli (19, 19%) and Staphylococcus aureus (nine, 9%) to be the most prevalent bacteria in their study. Interestingly, our current study accounted for only two (2%) of isolates of Gram-positive Staphylococcus aureus, suggesting a potential shift in the microbial spectrum over time, possibly due to evolving antibiotic pressures or local epidemiological factors. Findings may not be generalizable due to the single-center design and the exclusion of anaerobic and fungal cultures [17].

Additionally, no bacterial growth (30, 29.4%) was seen in a significant portion of the samples, which may be attributed to prior antibiotic use or the possibility of non-bacterial infections. A single case of polymicrobial infection (one, 1%) was also seen, which highlights the potential complexity of diabetic foot ulcers, where multiple pathogens may be involved.

The antibiotic sensitivity and resistance patterns observed in this study underscore the clinical challenges posed by multidrug-resistant organisms. Meropenem (n = 43, 83.7%), imipenem (n = 50, 74.0%), and piperacillin-tazobactam (n = 54, 72.2%) demonstrated the highest sensitivity among the antibiotics tested, reaffirming their utility in the empirical treatment of severe diabetic foot infections. Colistin and polymyxin B also proved effective against Pseudomonas aeruginosa, with sensitivity rates of 100% and 92.3%, respectively.

Conversely, co-amoxiclav exhibited a notably high resistance rate (n = 45, 86.5%), limiting its role in empirical therapy. Similarly, cefotaxime showed a high resistance rate (n = 34, 77.3%), supporting the need to avoid it as a first-line agent. Of particular concern is the resistance of Escherichia coli to carbapenems, including meropenem (n = 11, 91.7%) and imipenem (n = 10, 83.3%), which may suggest the presence of carbapenem-resistant Enterobacteriaceae (CRE). These pathogens are associated with increased morbidity, prolonged hospital stays, higher healthcare costs, and elevated mortality compared to carbapenem-susceptible strains [25].

The study underscores the importance of using local antibiograms to guide antibiotic therapy, given the significant variation in bacterial prevalence and resistance patterns compared to previous studies [16]. The data also highlight the need for cautious use of carbapenems and the importance of reserving colistin and polymyxin B for confirmed resistant cases due to their toxicity profiles.

Study limitations

This study has several limitations that must be acknowledged despite its important findings. The absence of a control group pre- and post-treatment analysis limits the ability to attribute changes in laboratory parameters solely to hospital-based treatment, as improvements may reflect natural variation or unmeasured confounders. Although the correlation between TLC and HbA1c was statistically significant, the small effect size indicates limited clinical predictive value. Additionally, the weak and non-significant correlation between ALC and Wagner grade, along with the cross-sectional study design, restricts the ability to establish causal relationships between immune markers, glycemic control, and ulcer severity.

Recommendations

To minimize the development of antibiotic resistance, strong antibiotics such as carbapenems and colistin should be reserved for cases where they are truly necessary. Physicians are encouraged to follow established antimicrobial stewardship guidelines to ensure appropriate selection and use of antibiotics. Additionally, diagnostic approaches should be expanded to include tests for organisms that may not grow in routine cultures, such as anaerobic bacteria and fungi, particularly when initial cultures yield no growth. It is also essential to educate diabetic patients on proper foot care and recognizing early signs of infection to prevent complications that could necessitate the use of broad-spectrum or last-resort antibiotics.

## Conclusions

This study highlights the relationship between immune markers, glycemic control, bacterial resistance, and diabetic foot ulcer severity. While ALC and Wagner grade were not reliable predictors, poor glycemic control was linked to higher TLC and slower healing. Treatment reduced blood sugar and inflammation but had little effect on ALC, hemoglobin, and platelets. A shift toward more Pseudomonas aeruginosa and Escherichia coli, along with rising antibiotic resistance, was observed. This multidisciplinary approach improves understanding of DFU severity and outcomes, though further large-scale studies are needed.
